# Prevalence of color vision deficiency among school children in Wolkite, Southern Ethiopia

**DOI:** 10.1186/s13104-018-3943-z

**Published:** 2018-11-28

**Authors:** Gashaw Garedew Woldeamanuel, Teshome Gensa Geta

**Affiliations:** Department of Biomedical Sciences, School of Medicine, College of Medicine and Health Sciences, Wolkite University, P.O. Box 07, Wolkite, Ethiopia

**Keywords:** Colour vision deficiency, Prevalence, School children

## Abstract

**Objective:**

Colour vision deficiency is the commonest disorders of vision and undiagnosed colour vision defect could pose a handicap to the performance of an affected student. The prevalence of colour blindness varies in different geographical area and ethnicity. Hence, a cross sectional study was conducted among school children in Gurage Zone, Southern Ethiopia from April 15 to June 20, 2018. Socio-demographic data was collected on a face to face interview using structured questionnaire. All study participants underwent color vision evaluation using Ishihara’s pseudo isochromatic test 38 plate editions. Data analysis was done using SPSS version 23.

**Results:**

A total of 844 (471 boys and 373 girls) school children were screened for colour vision. The overall prevalence of colour vision deficiency was 4.1%, comprised of 3.6% in boys and 0.6% in girls. Out of 35 color blind subjects, 15 (42.9%) and 20 (57.1%) were the victims of protan and deutan defects respectively. Majority of the colour blind subjects were not aware of their colour vision status. Hence, the study concluded that the prevalence of colour vision deficiency in our study is significant and colour vision screening among school should be performed.

## Introduction

Colour vision deficiency (CVD) or colour blindness, is the inability or decreased ability to distinguish different colours under normal lighting conditions [1]. It is one of the commonest disorders of vision [2] and the incidence of CVD varies from race to race and different in different geographical area [3, 4]. However, most of colour blind cases remain undetected due to absence of proper screening [5].

Colour vision deficiency may be congenital or acquired. Congenital colour defects are non pathologic, incurable, and constant throughout life [6]. It is the commonest X- linked recessive disorder and affects as many as 8% of males and 0.5% of females [2]. There are also different causes for acquired colour vision defects, such as ocular or neurological disease, some metabolic disorders, drug toxicity and exposure to certain solvents [5, 7].

Alterations of the three (red, green and blue) cone pigments are responsible for colour vision deficiencies. The commonest form of deficient colour vision is red-green deficiency [8, 9], in which either of the red or green cones is missing. The prevalence of red- green colour deficiency in European Caucasians is about 8% in men and about 0.4% in women and between 4% and 6.5% in men of Chinese and Japanese ethnicity. The prevalence is also rising in men of African ethnicity [10].

Colour vision deficiency does not cause complete blindness and there is no available therapeutics that can treat CVD [11]. However, colour vision is crucial to an individual’s understanding of their visual world, and those with colour vision defects can experience difficulties in everyday life [5]. Those who have CVD will be better able to adapt and make more informed career choices, if they know about their colour vision status. However, a high proportion of school children are unaware of their colour vision status and undiagnosed CVD could pose a handicap to the scholarly performance of an affected student [12]. Moreover, early detection of colour vision malfunction in children allows parents and teachers to make necessary adjustments to the teaching methods for appropriate learning [13]. There is only limited report in Ethiopia and no such report in the southern Ethiopia about the prevalence of colour blindness among both sexes of school children. Hence, the aim of this study was to determine the prevalence of colour vision deficiency among school children in Wolkite town, Southern Ethiopia.

## Main text

### Methods and materials

#### Study area and sampling

School based cross sectional study was conducted in Wolkite town, Southern Ethiopia from April 15 to June 20, 2018. Wolkite town is located at about 158 km from the capital city, Addis Ababa. In the year 2018, the town had a total of 12 primary schools hosting approximately 13,510 (6820 boys and 6690 girls) students from grade 1–8.

The source populations of the study were all primary school children in Wolkite town. School children, who met the inclusion criteria from the target, were selected as the participants of this study. An inclusion criterion for this study was all primary school children who had a written consent from the parents or guardians. Children with any evidence of ocular pathology, trauma, previous ocular surgery, long term use of medication and students born to parents who are not from Ethiopia were excluded from the study.

The required sample size was calculated using statistical formula for single population proportion by considering the proportion of defective colour vision in the previous study as 4.2% [14], with 95% confidence interval, 2% margin of error, design effect of 2 and with the assumption of 10% non response rate. Hence, the final sample size was 850.

The study participants were selected through multistage sampling technique. Initially, 3 schools out of 12 schools were selected randomly using lottery method. Then, the total sample size was allocated to the selected schools based on probability proportional to size sampling. Finally, a systematic random sampling was employed to select study participants. The student lists was obtained from the school director office and sampling fraction was calculated for each school and it was 5. The first sample student was selected from 1 to 5 of the student list by lottery method and then the remaining possible sample students were selected every 5 students.

#### Data collection procedure

The data were collected by ophthalmic nurses on a face to face interview using structured questionnaires. After collection of socio-demographic characteristics, all the study subjects underwent colour vision evaluation. Colour vision test was done using Ishihara pseudoisochromatic plates (38 plates edition). Before performing the test, the procedure was clearly explained to all subjects. Then, the test was conducted in a room with adequate natural daylight. The plates were held 75 cm from the study subject and the students were asked to read the numerals which are seen on plates within 3 s. The results were classified based on the instructions described in the attached manual of Ishihara’s test plates [15]. Overall, the test was conducted based on the standard recommendation of colour vision test and the details of test process have been described previously [1, 14]. Children having colour vision deficiency underwent detail ocular examination to rule out any ocular pathology. Data quality was assured via training of data collectors, supervision of data collection and the questionnaires were pre tested.

#### Data analysis

Data were entered into CSPro version 6.2 statistical software and then exported to SPSS version 23 for analysis. Descriptive statistics was used to present the socio-demographic characteristics of the study participants. Chi square (χ^2^) test was used to assess statistical significances and p-values of less than 0.05 were considered as statistically significant.

### Results

A total of 844 (471 boys and 373 girls) children from 3 randomly selected primary schools were screened with a response rate of 99.3%. The mean (± standard deviation) age of participants was 11.75 (± 2.5) years and the majority of participants were Muslim by religion (Table 1).

**Table 1:** Socio-demographic characteristics of school children in Wolkite, Southern Ethiopia, 2018 (n = 844)

Variables	n (%)
Sex	
Male	471 (55.8)
Female	373 (44.2)
Age (years)	
7–12	493 (58.4)
13–18	351 (41.6)
Religion	
Muslim	387 (45.9)
Orthodox	364 (43.1)
Protestant	46 (5.5)
Catholic	47 (5.6)
School grade	
1–4	393 (46.6)
5–8	451 (53.4)
School	
Ras Zesilassie	355 (42.1)
Aba Firansua Markos	310 (36.7)
Gasora and Karacha	179 (21.2)

**Fig. 1:**
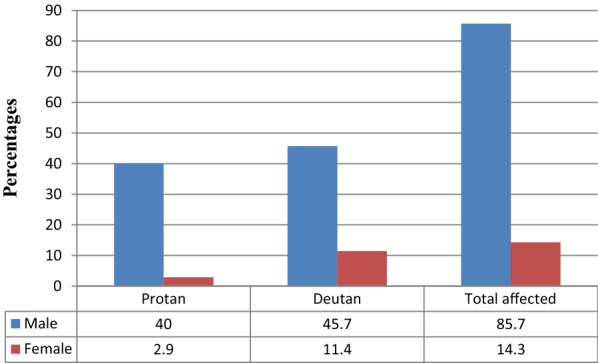
Distribution of the colour blind subjects according to different types of Color blindness in Wolkite, Southern Ethiopia, 2018 (n = 35)

After careful screening, it was noted that among the study participants, 35 (4.1%) students were found to have colour deficient. Out of those students with CVD, 15 (42.9%) and 20 (57.1%) were classified as having protan and deutan defects, respectively. Hence, the deutan to protan ratio was 1.3. The prevalence of protan and deutan defects were higher among males as compared to female children (Fig. 1). In the current study, no case of total colour blindness was detected. Moreover, none of the enrolled subjects showed evidence of ocular pathology. Majority, 32 (91.4%) of the CVD students were unaware of their defect.

In this study, the gender based differences in the prevalence of color vision deficiency was found to be statistically significant (p < 0.001), with a higher prevalence among male (3.6%) as compared to female (0.6%) students. Similarly, there was statistically significant difference in CVD across religion (p = 0.039) in which Muslim children had a higher percentage of CVD than others (Table 2).

**Table 2:** Distribution of students according to the color vision status among school children in Wolkite, Southern Ethiopia, 2018

Variables	Color vision		χ^2^	p-value
	Normal (%)	Deficient (%)		
Sex				
Male	441 (93.6)	30 (6.4)	13.24	*< 0.001*
Female	368 (98.7)	5 (1.3)		
Age (years)				
7–12	478 (97)	15 (3)	3.64	0.057
13–18	331 (94.3)	20 (5.7)		
Religion				
Muslim	365 (94.3)	22 (5.7)	4.25	*0.039*
Others	444 (97.2)	13 (2.8)		
School grade level				
1–4	374 (95.2)	19 (4.8)	0.87	0.350
5–8	435 (96.5)	16 (3.5)		
Medical visit				
Yes	148 (93.7)	10 (6.3)	2.33	0.127
No	661 (96.4)	25 (3.6)		

Numerical data in italic indicates the level of significance (p < 0.05), χ^2^** = **Chi square

### Discussion

This study provides a detailed description of colour vision deficiency in the study area for the first time among school children which provides a basic database on the prevalence of colour blindness in the region. Screening for colour vision in children should be performed for early detection of colour vision deficiency and to reduce the risk of difficulties in lifestyle. In the present study, no other acquired causes of CVD were found. Thus, the possible mechanism of colour blindness might be congenital. A similar finding was also reported by other study [16].

Total colour blindness is the severest forms of congenital colour vision deficiency and was not detected in our study. Hence, colour blind children of this study were partially blind. In the present study, the prevalence of colour vision deficiency was 4.1%, which is nearly similar with the prevalence rates reported in the previous studies [11, 14, 17]. This finding was also comparable with a study done in Thailand, which reported a prevalence rate of 4.2% [18]. However, the result of this study is higher than the incidence of colour blindness among school children reported as 1.9% [3] and 2.1% [19] in Nepal, 2.6% in Nigeria [20] and 1% in Bangladesh [16]. A higher prevalence of 5.28% was reported in India among Manipuri Muslims of both sexes [5]. These variations in the prevalence of CVD from the current study could be due to difference in study population, variation in geographical area, ethnicity and techniques of color vision test.

In the present study the percentage of colour vision deficiency was higher among boys (3.6%) as compared to girls (0.6%). This finding is supported by several studies [1, 13, 20, 21]. While other studies [3, 16, 19] showed that colour blindness was found only among the males. Although, the male: female prevalence ratio is markedly different in different studies, several published literatures showed a higher prevalence of CVD among males compared to females. This is due to X-linked recessive nature of the trait and thus occurs in males [5].

The present study also revealed that most of the colour blind children were deutan. In line with this finding, a high frequency of deutan as compared to protan defects was observed in other studies [1, 14, 22].

Study done by Bowmaker [23] indicated that the most common form of anomalous colour vision is deuteranomaly. It was suggested that green colour receptor is commonly affected than other cone receptors [16, 23].

Majority of the colour blind children of present study were Muslim by religion, which is supported by other study [16]. Colour blindness is common among the Muslim populations due to frequently practice of consanguinity of marriage among them [16]. Similarly, study done in India [5] and in young Turkish men [24] showed that higher percentages of colour blindness were found in regions with a more consanguineous marriages, which might result in the birth of children with this disorder.

In our study, majority of the study participants did not have a chance of eye examination at all. Students must be made aware of their congenital colour vision deficiency but majority of the CVD students were unaware of their colour vision status. In agreement to our study, study done by Mulusew and Yilkal [14] reported that almost all of the study subjects were not aware of their colour vision status. Hence, colour vision screening among school children should be conducted to increase the level of awareness about their colour vision defects.

### Conclusion

The prevalence of colour vision deficiency among school children in the study area was 4.1%. The percentage of colour vision deficiency was found higher among males as compared to females. Children awareness of colour vision status was found to be very low. This indicates the need for establishing continuous visual screening programs among school children. As a recommendation, further community based screening of children for colour vision should be done.

## Limitations

Anomaloscope should be used for detailed analysis of quantitative and qualitative anomalies in colour perception. We couldn’t use anomaloscope in our study because the instrument was not available in our set up. The choice of school rather than population based sample was also another limitation of this study. Despite the above limitation this study provides valuable information about the burden of colour vision deficiency among school children in Wolkite town.
